# Protective effects of essential oil from Fructus *Alpiniae zerumbet* on retinal Müller gliosis via the PPAR-γ-p-CREB signaling pathway

**DOI:** 10.1186/s13020-019-0283-4

**Published:** 2020-01-10

**Authors:** Hong Yang, Shiquan Gan, Zhaohui Jiang, Xiaomei Song, Tingting Chen, Yini Xu, Lingyun Fu, Yanyan Zhang, Ling Tao, Xiangchun Shen

**Affiliations:** 1 The Department of Pharmacology of Materia Medica (the State Key Laboratory of Functions and Applications of Medicinal Plants, the High Educational Key Laboratory of Guizhou Province for Natural Medicinal Pharmacology and Druggability), School of Pharmaceutical Sciences, Guizhou Medical University, University Town, Guian New District, Guizhou, 550025 China; 2 The High Efficacy Application of Natural Medicinal Resources Engineering Center of Guizhou Province (the Union Key Laboratory of Guiyang City-Guizhou Medical University), School of Pharmaceutical Sciences, Guizhou Medical University, University Town, Guian New District, Guizhou, China; 3 The Key Laboratory of Optimal Utilization of Natural Medicine Resources, School of Pharmaceutical Sciences, Guizhou Medical University, University Town, Guian New District, Guizhou, China

**Keywords:** Essential oil of Fructus *Alpiniae zerumbet*, Retinal Müller cells gliosis, Diabetic retinopathy, PPAR-γ, CREB

## Abstract

**Background:**

Diabetic retinopathy (DR) involves extensive retinal damage and is one of the most common and serious complications of diabetes mellitus. Hyperglycemia is the major pathological trigger for diabetic complications. Müller cell gliosis, a key pathophysiological process in DR, could finally lead to vision loss. Our previous finding revealed that the essential oil of Fructus *Alpiniae zerumbet* (EOFAZ) protects human umbilical vein endothelial cells (HUVECs) against high glucose (HG)-induced injury via the PPAR-γ signal. However, Whether EOFAZ could prevent HG-induced Müller cell gliosis through the PPAR signaling remains unclear.

**Methods:**

The neuroprotective effects of EOFAZ were evaluated in HG-treated rat retinal Müller cells (RMCs) and DR rat model.

**Result:**

GFAP and VEGF upregulation is the biomarker of Müller glial reactivity gliosis. Results suggested that EOFAZ could remarkably ameliorate retinal reactive gliosis by suppressing p-CREB and GFAP and VEGF downstream effectors. Its effects on PPAR-γ, a major target for currently available anti-diabetes drugs, were also investigated. EOFAZ treatment remarkably attenuated the reduction of PPAR-γ and high level of p-CaMK II and p-CREB in HG-treated RMCs and diabetic rats. Furthermore, the activation and ectopic expression of PPAR-γ downregulated p-CREB and p-CaMK II in HG-treated RMCs. By contrast, CaMK II inhibitor KN93 and CREB gene silencing did not significantly affect the PPAR-γ expression.

**Conclusions:**

A novel PPAR-γ-p-CREB signaling pathway accounts for the inhibitory effect of EOFAZ on RMCs gliosis. These findings provide scientific evidence for the potential use of EOFAZ as a complementary and alternative medicine for DR prevention and treatment in the future. 
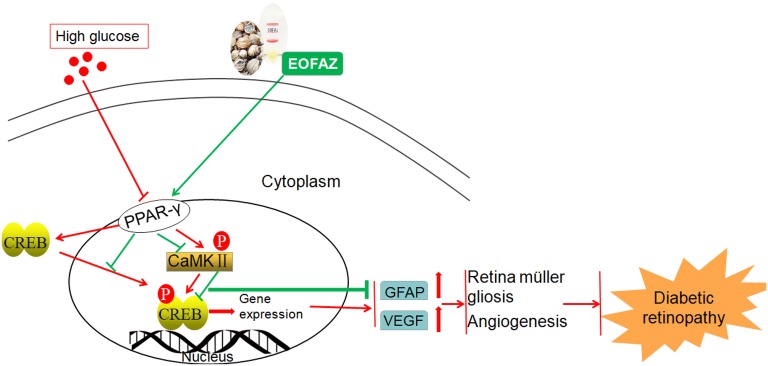

## Background

With its increasing incidence, type 2 diabetes (T_2_DM) has become an epidemic that affects populations at different life stages, and is considered a heavy global burden. Diabetic retinopathy (DR) is a common microvascular complication of diabetes, and the leading cause of new-onset vision loss in adults worldwide. Many pathological factors, such as hyperglycemia and metabolic dysregulation, are involved in this process. Normal retinal function is maintain through neurovascular interactions, and any impairment could be the key event in DR pathogenesis [1]. This complication involves alterations in the microvasculature, neurons, and glia. A disorder of neurovascular interactions is associated with neovascularization in the retina, and neovascularization is a DR hallmark. Accumulating pieces of evidences confirmed that vascular endothelial growth factor (VEGF) overexpression is involved in vascular development during retinal angiogenesis [2], thereby leading to severe vision loss and irreversible blindness [3, 4].

Müller glia is the main glia in retinas and their source of VEGF secretion. These cells span the entire depth of the retina, provide structural and trophic support to surrounding cells, and link and support retinal blood vessels and neurons to form the retinal vasculature. Glial cells become reactive following any retinal damage or disruption of homeostasis, and this response is termed reactive gliosis. Gliosis is a feature of several retinal diseases, including DR. Müller glia is a major contributor retinal gliosis [5]. Reactive gliosis in retina is induced by the over-proliferation of retinal Müller cells (RMCs), and may trigger neuronal cell death via glial scar formation [6].

Glial fibrillary acidic protein (GFAP) upregulation is the biomarker of Müller glial reactivity gliosis [7]. A high VEGF level is observed in the development of glial gliosis. VEGF and GFAP are overexpressed in RMCs under hyperglycemic conditions [8], and are therefore considered as the key regulator factors in retinal Müller gliosis and eventually in DR.

cAMP response element binding protein (CREB) is a ubiquitous nuclear factor implicated in neuronal survival. CREB could be phosphorylated at Ser-133 by CaMK II, a member of the CaMK family [9]. p-CREB, the activated form of CREB, binds to DNA and regulates the transcription of various target genes, such as GFAP [10, 11].

PPAR-γ, a member of the peroxisome proliferator activated receptor (PPAR) subfamily of nuclear receptors, has a decreased level in the retina of diabetes animal models. And activation of its homolog, PPAR-α, is beneficial to DR [12]. However, the protective effect of PPAR-γ in DR, especially in reactive gliosis, is poorly understood. Several studies have implied the neuroprotective effects of PPAR-γ, for example, PPAR-γ agonist thiazolidinediones could attenuate reactive gliosis [13]. Rosiglitazone (RGZ), a specific PPAR-gamma ligand, exerts anti-angiogenic activity by regulating retina cell proliferation and VEGF expression as a normal route for DR treatment [14]. Therefore, RGZ was chosen as the positive drug in the present study. PPAR-γ inhibition modulates p-CREB and its nuclear translocation [10], suggesting the crosstalk between PPAR-γ and CREB in retinal gliosis.

Phytotherapy is a highlighted as a treatment for escalating epidemics such as vascular diseases and diabetes [15]. The ethanol extract of *Zerumbet rhizome* (*L*) Smith can prevent DR [16]. However, whether extracts of other species of Zingiberaceae have similar potential therapeutic effects on DR remains unknown. Fructus *Alpiniae zerumbet* is used as a folk medicinal herb by the Miao people in the Guizhou Province of China. Our previous study indicated that essential oils from Fructus *Alpiniae zerumbet* (EOFAZ) have protective effects against high glucose-induced human umbilical vein endothelial cell injury [17], can ameliorate inflammation, and inhibit ox-LDL-induced oxidative stress [18, 19]. In addition, 1,8-cineole, one of the main active compounds of EOFAZ, can stimulate PPAR-γ [20]. However, detailed mechanisms underlying the protective effects of EOFAZ on HG-induced retina injury, especially retinal gliosis, remain unclear. The signaling mechanism for retinal gliosis should be elucidated to discover novel therapeutic approaches to DR.

Our findings highlight on the neuroprotective effects of EOFAZ on RMCs gliosis and provide evidences that EOFAZ can ameliorate DR. and may be beneficial for DR prevention and treatment in the future.

## Materials and methods

### Materials and reagents

The Fructus *Alpiniae zerumbet* (FAZ) was collected from Zhenfeng County, Guizhou Province, China. Streptozocin (STZ) was purchased from Sigma (St Louis, MO, USA). RGZ, GW9662 and KN93 were obtained from Sigma (St Louis, MO, USA) and Selleckchem (Shanghai, China), respectively. Commercial kits, measuring insulin, VEGF and blood glucose, were obtained from Elabscience Co. Ltd. (Shanghai, China), Xin Bo Sheng (ERC103, China), Yuanye company (Shanghai, China), respectively. The primary anti-bodies used in this study including anti-GFAP, anti-Phospho-CREB, anti-CREB, anti-Phospho-CaMK II, and anti-CaMK II were supplied by Cell Signaling Technology (Danvers, MA, USA); anti-PPAR-γ, anti-VEGF and anti-β-actin were purchased from Proteintech (Chicago, USA). All reagents used for qRT-PCR were obtained from Takara Bio. (Dalian, China). PPAR-γ siRNA and CREB siRNA were supplied by GenePharma (Shanghai, China).

### Extraction of essential oil from Fructus *Alpiniae zerumbet*

The Fructus *Alpiniae zerumbet* (FAZ) was authenticated by associate Professor Qing-De Long, and the voucher specimen (No. 20151018) was deposited at the Key Laboratory of Optimal Utilization of Natural Medicine Resources, the School of Pharmaceutic Sciences, Guizhou Medical University (Guizhou, China). As previously described, the essential oil was extracted from FAZ by steam distillation technology [18]. Then, the essential oil was dried using anhydrous sodium sulfate and stored at − 20 °C. The total yield is around 1.3%. The composition of the essential oil was determined by gas chromatography and mass spectrometry, including α-Pinene, Camphene, β-Pinene, β-Myrcene, o-Cymol, β-Phellandrene, 1,8-Cineole, Linalool, Camphor, (−)-Borneol, 4-Terpineol, (−)-α-Terpineol, trans-Caryophyllene, Nerolidol, Caryophyllene oxide, α-Cadinol, t-Muurolol. Obtained EOFAZ was dissolved in DMSO (10 mg/mL) storage at 4 °C.

### Animal model establishment and maintenance

Three-month-old male Sprague–Dawley (SD) rats were purchased from Guizhou Medical University Laboratory Animal Co., Ltd. (Guizhou, China). All procedures were performed in accordance with the recommendations of the Guide for the Care and Use of Laboratory Animals of the National Institutes of Health. All rats were maintained in a pathogen-free facility (23 °C ± 2 °C, 50% ± 15% relative humidity, under a 12:12 h light/dark cycle) with free access to water and regular chow or high fat and high sucrose diet (HFSD). For fasting experiments, rats were fasted for 16 h from 6:00 pm to 10:00 am.

Type 2 diabetic rat model was induced as previously described [21]. After 12 weeks, serum samples were collected from the tail veins. Fasting serum indices, including serum triglycerides (TGs), serum cholesterol (TC) and the fasting blood glucose were detected using an automatic biochemical analyzer, The fasting insulin levels were analyzed by ELISA. And analyzed the homeostasis and generate insulin resistance index (HOMA-IR) of rats.

The HFSD-fed rats were rendered diabetes by intraperitoneal (IP) injection of streptozotocin (STZ, 25 mg/kg body weight) at week 12. After 1 week of STZ injection, the blood glucose levels were consistently above 16.7 mM, and the model was considered successful, The rats were then given regular diet and randomly divided into two groups as following: the control group (n = 8, normal saline, intragastrically [i.g.] administrated) and EOFAZ group (n = 8, 0.18 g/kg, i.g, dissolved in 0.5% Tween 80, diluted with normal saline), to analyze the effect of EOFAZ on the physiological function of normal rats. Diabetic rats were randomly allocated into two groups as follows: STZ + DM group (n = 8) and STZ + DM + EOFAZ groups (n = 8). A week after STZ injection, the rats in the EOFAZ group and the STZ + DM + EOFAZ group were administrated with EOFAZ when rats in control group and STZ + DM group were given saline for the next 8 weeks. Serum sample was obtained from the tail veins to assay the fasting blood glucose levels weekly in the last 8 weeks. Body weight was recorded every week.

### Histological analysis

The eye was collected, and then fixed with 4% paraformaldehyde in phosphate buffer saline. The paraffin sections (4 μm) were stained with hematoxylin and eosin (H&E) for histological evaluation. The pathological images were obtained by microscope at 400× magnifications under an optical microscope (Leica, Germany), respectively. Retinal damage was evaluated by hemorrhage and changes structure of retinal difference layers, including outer nuclear layer (ONL), outer plexiform layer (OPL), Inner nuclear layer (INL) and ganglion cell layer (GCL).

### Immunohistochemistry

Immunohistochemistry was performed as previously described [22], tissue blocks were fixed in 10% buffered neutral formalin, and embedded in paraffin wax, paraffin-embedded tissue blocks were continuously sectioned in 5 μm thickness. The sections were subject to antigen retrieval in citrate buffer and then incubated overnight at 4 °C with the primary antibodies (GFAP, VEGF) followed by secondary antibody (PV-6000) for 30 min at room temperature subsequently. The slides were washed again in PBS and incubated with 0.01% 3,3-diaminobenzidine tetrahydrochloride (DAB) for approximately 10 min. Then, the samples were counterstained with haematoxylin for 20 s, dehydrated in absolute alcohol and cleared in xylene, and finally mounted in synthetic resin. The digital images of the samples were observed and obtained by microscopy (Leica, Germany).

### ELISA

Individual blood sample was taken from the tail vein, sera were separated without anticoagulant, and aliquoted for analysis. All experimental assays were operated in accordance with the manufacturers’ instructions. All samples was analyzed in triplicate.

### Cell culture and treatment

Rat Müller glial-derived cell line (RMCs, Saiqi Biological Engineering Co., Ltd, Shanghai, China) were cultured in Dulbecco’s modified Eagle’s medium (DMEM) supplemented with 10% FBS and 1% penicillin/streptomycin. Cell culture was maintained in the atmosphere of 95% humidity and 5% CO_2_ at 37 °C. RMCs proliferation model reproduced by high glucose (HG, 30 mM) for 48 h, and 5.5 mM glucose medium as the control. To explore the effect of EOFAZ on RMCs challenged by HG, RMCs were pre-treated with EOFAZ, RGZ, GW9662 and KN93 for 1 h, then directly exposed to HG in 1.5% FBS for 48 h. Cells in the control group were incubated with DMEM with 1.5% FBS for the next 48 h. The mannitol group (24.5 mM) was used to exclude the osmosis impact on RMCs.

### Cell proliferation assay

Cell viability was measured by MTT assay. Briefly, RMCs (5 × 10^3^ cells/well) were cultured in 96-well plates until grown to 80% confluence. Briefly, cells were exposed to HG (30 mM) for 48 h, the culture medium was completely removed, and incubated the cells with 180 μL fresh medium and 20 μL MTT (5 mg/mL) for 4 h, the medium was replaced with 150 µL dimethyl sulfoxide solution (DMSO), and the spectrometric absorbance (490 nm) was measured by ELX800 microplate reader (Bio Tek, USA).

### Flow cytometry analysis for cell cycle

RMCs were seeded into six-well plates. The cells were treated for 48 h and fixed in 75% methanol at 4 °C overnight. The cells were washed twice with cold PBS and stained with PI (0.1 mg/mL plus 0.5 mg/mL RNase in PBS) for 30 min at room temperature. The stained cells were characterized by flow cytometry (NovoCyte 3008), DNA content and cell cycle were analyzed by FlowJo software.

### Giemsa staining

RMCs were stained with Giemsa solution to assess the pathological morphological characterization of RMCs. The images were visualized under an inverted microscope (200×).

### Transient transfection

Over-expression PPAR-γ plasmid was generously provided by Department of Pharmacology, Nanjing Medical University (Nanjing, Jiangsu, China). siRNA (small interfering RNA) targeting PPAR-γ and CREB were designed and synthesized by GenePharma (Shanghai, China). Briefly, RMCs were seed into 6-well plate at 30–50% confluence. After 24 h, RMCs were incubated with Lipofectamine 2000 reagent and siRNAs in accordance with the manufacturer’s protocol. The transfected cells were collected 48–72 h later.

### Quantitative real-time PCR (qRT-PCR)

Total RNA was extracted from RMCs by the TaKaRa MiniBEST universal RNA extraction kit (TaKaRa, Dalian, China) according the manufacturer’s protocol. mRNA was reverse-transcribed into cDNA using the Reverse Transcription Kit. Real-time PCR was performed on a Bio-Rad Real-Time PCR System (Applied Biosystems Co., Foster City, United States). cDNA was amplificated using SYBR Green PCR reagents (TaKaRa) with primers specific for amplification of PPAR-γ, VEGF, GFAP and β-actin. Analysis was performed using the comparative cycle threshold method (2^−ΔΔCt^), and target gene expression was normalized to the expression of β-actin. Primer sequence is show in Table 1.

**Table 1:** Specific primers for quantitative RT-PCR

Gene	Forward	Reverse
PPAR-γ	CGATGCTGTCCTCCTTGATG	GCAGAATGGCTTCCTCAGGT
VEGF	CGGAGAGCAACGTCACTATG	GGTCTGCATTCACATCTGCT
GFAP	CCTGAGGCAGAAGCTCCAAG	AGATCCACACGAGCCAAGGT
β-Actin	TGTCACCAACTGGGACGATA	GGGGTGTTGAAGGTCTCAAA

### Western blot

RMCs and the whole retina were homogenized in RIPA buffer (Beyotime Biotechnology) containing a protease inhibitor and a phosphatase inhibitor (Kang Chen). Total proteins (30–50 μg) were separated by 10% SDS-PAGE gel (solarbio) and transferred to PVDF membrane (immobilon-P^SQ^). The membranes were blocked with 5% BSA in TBST buffer for 1 h, then incubated overnight at 4 °C with primary antibody followed by HRP-conjugated secondary antibody. Digital images of blots were obtained from a Syngene Gel Imaging System (Bio-Rad) and quantified using Syngene software. All antibodies and dilution ratio were listed in Table 2.

**Table 2:** Specific antibodies for Western blot

Antibody	Company	Catalogue number	Dilution
GFAP	CST	12389	1:1000
VEGF	Proteintech	19003-1-AP	1:1000
PPAR-γ	Proteintech	16643-1-AP	1:3500
Phospho-CREB	CST	9198	1:1000
CREB	CST	9197	1:1000
Phospho-CaMKII	CST	12716	1:1000
CaMKII	CST	3362	1:1000
β-Actin	Proteintech	20536-1-AP	1:3500

### Statistical analysis

Data are the mean ± SD of at least three independent experiments. Data were statistically analyzed using Student t-test or two-way ANOVA with multiple comparisons in GraphPad Prism 6 software. *P* < 0.05 was considered statistically significant.

## Results

### Effects of EOFAZ on metabolic parameters in rats

**Fig. 1:**
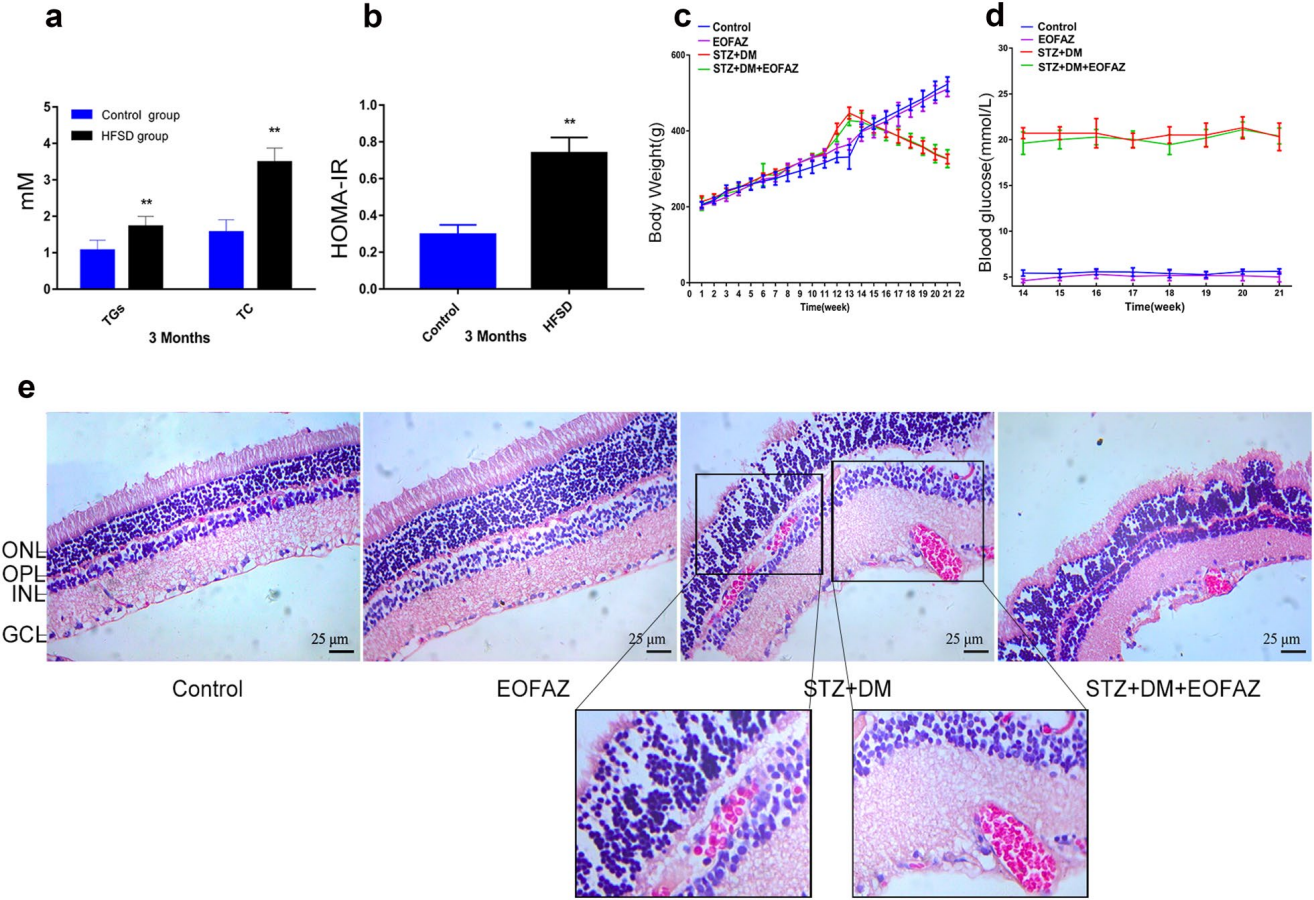
Effects of EOFAZ on metabolic parameters and retina histology in diabetes rats. Rats were daily fed with HFSD for 3 months. Detection of TGs and TC in serum (**a**). Analysis of HOMA-IR index (**b**). Body weight level was recorded every week throughout the study (**c**). The rats were rendered diabetic via IP injection of STZ (25 mg/kg body weight), and blood glucose levels were recorded after diabetes rats were supplemented with EOFAZ during the last 8 weeks (**d**). After hematoxylin–eosin staining, the pathological images of sections from the retina were taken under a microscope at ×40 magnification (**e**). *ONL* outer nuclear layer, *OPL* outer plexiform layer, *INL* inner nuclear layer, *GCL* ganglion cell layer. Data are expressed as mean ± SEM, n ≥ 6, **P < 0.01 vs. non-diabetic rats

Rats were daily fed with HFSD for 3 months, and blood samples were collected from the tail vein to detect serum TGs and TC levels, as shown in Fig. 1a. The TGs and TC levels of rats fed with HFSD were significantly higher than the normal diet-fed rats. Fasting blood glucose and insulin levels were detected to analyze the HOMA-IR of rats to monitor the insulin resistance of T_2_DM rats. As shown in Fig. 1b, the HOMA-IR index in the HFSD-fed rats was significantly higher than that of the normal diet-fed rats, HFSD-fed rats displayed insulin resistance and successful establishment of T_2_DM rat model. HOMA-IR was calculated with (fasting insulin [mIU/L] × fasting glucose[mmol/L])/22.5.

The HFSD-fed rat was rendered diabetic via the intraperitoneal injection of STZ at week 12. As shown in Fig. 1c, the body weight of diabetic rats increased from week 1 to 12, but decreased from week 14 to 21. However, the body weight of non-diabetic rats kept increasing from week 1 to 21. The fasting blood glucose levels of diabetic rats were still significantly higher than those of non-diabetic rats. EOFAZ (weeks 14–21) did not alleviate decrease in the body weight of diabetic rats (Fig. 1c). Furthermore, the blood glucose level of STZ + DM + EOFAZ group was not significantly different compared with that of STZ + DM group (Fig. 1d). These data suggested that the effects of EOFAZ are not relative to weight and blood glucose control.

### Effects of EOFAZ on histology of retina in diabetic rats

As shown in Fig. 1e, the retinal layers of the control group had clear structure with tight and tidy cells. The retinal layers (ONL, OPL, INL and GCL) smoothness and integrity remain. GCL formed a single layer. However, OPL and GCL with edema and hemorrhage were discerned in the diabetic rats, and the EOFAZ-treated group obviously ameliorated abnormal morphological changes and relieved hemorrhage. Disrupted and disorganized INL and ONL were observed in diabetic rats. Administering EOFAZ dramatically improved the disorder and disarrangement in the diabetic rat retina. These results indicated that EOFAZ significantly ameliorates the pathological changes in the retina of diabetic rats.

### Effects of EOFAZ on retinal Müller gliosis in diabetic rats

**Fig. 2:**
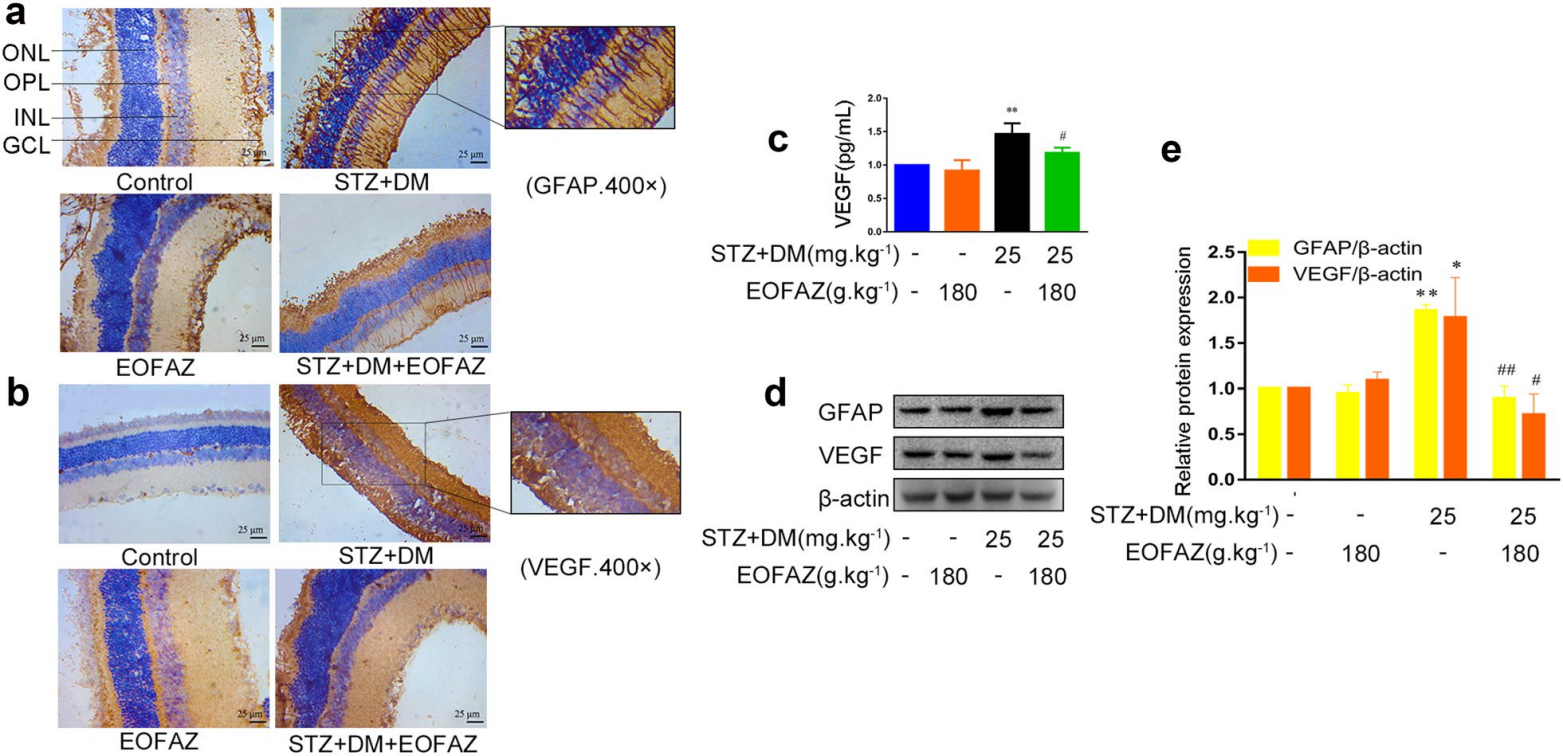
EOFAZ inhibited retinal Müller gliosis in diabetic rats. After Immunohistochemical staining to GFAP and VEGF, the morphological images were taken under a microscope (**a**, **b**). Scale bar, 25 μm. ONL: Outer nuclear layer, *INL* inner nuclear layer, *GCL* ganglionic cell layer, *OPL* outer plexiform layer. Detection of VEGF level in serum (**c**), n ≥ 6. The expression of GFAP and VEGF in retina was detected by Western blot (**d**, **e**), and quantitative analysis via densitometry and was expressed as relative to β-actin. *P < 0.05, **P < 0.01 vs. non-diabetic rats,^#^*P* < 0.05, ^##^*P *< 0.01 vs. diabetic rats

Müller glia is vulnerable to retina injury, and its subtle damage caused by high glucose levels initiates Müller gliosis. The abnormal upregulation of GFAP and VEGF is the hallmark of reactive gliosis in the retina [14, 23]. Our results are consistent with previous data, suggesting that GFAP and VEGF expression was increased in the whole retina of diabetic rats (Fig. 2), Thus, the reactive gliosis caused by diabetes, is enhanced. GFAP and VEGF expression levels were close to normal level after treatment with EOFAZ. Furthermore, the VEGF level in the serum of rats was detected by ELISA to further confirm that the ameliorated effect of EOFAZ on gliosis in diabetic rats. As shown in Fig. 2c, the diabetic rats exhibited considerably higher levels of VEGF than non-diabetic rats. After treatment with EOFAZ for 8 weeks, EOFAZ resulted in profound retina protection by reducing the diabetes-induced increase of VEGF in serum (Fig. 2c). Thus, EOFAZ can reduce retinal Müller gliosis in diabetic rats by suppressing GFAP and VEGF expression in the retina.

### EOFAZ improved HG-induced gliosis in RMCs

**Fig. 3:**
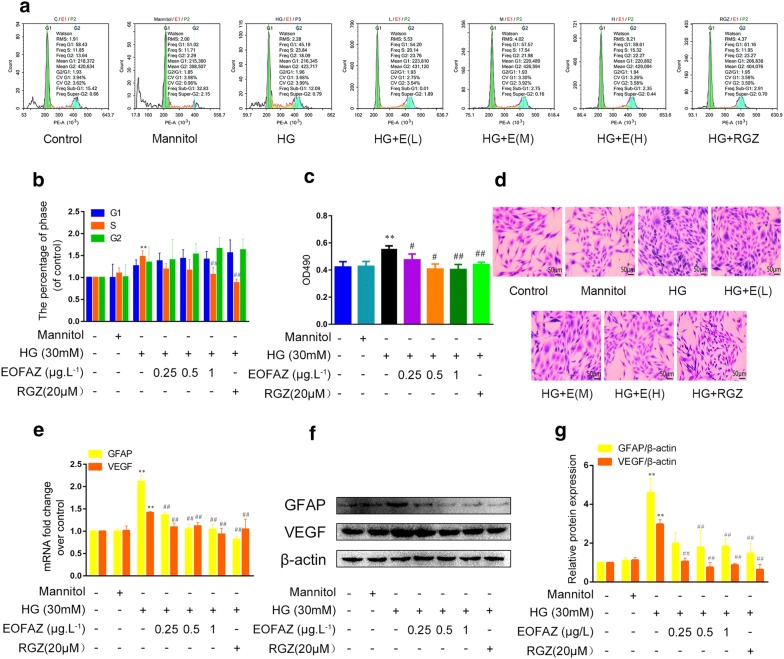
EOFAZ ameliorated RMCs gliosis induced by HG. RMCs were treated with 30 mM HG in combination with EOFAZ (0.25, 0.5 and 1 μg/L) for 48 h. The percentage of RMCs in each phase of the cell cycle, including G1, S, and G2 phase, was detected by flow cytometry (**a**, **b**). Cell viability was analyzed via the MTT assay (**c**). Giemsa staining was used to analyze the pathological morphology in RMCs (×200) (**d**). qRT-PCR (**e**) and Western blot (**f**, **g**) were used to analyze mRNA or protein expression of GFAP and VEGF, respectively. Data are expressed as mean ± SEM. n = 3. **P < 0.01 vs. control group, ^#^*P *< 0.05, ^##^*P *< 0.01 vs. HG treated group

Chronic hyperglycemia is one of the direct pathological factors inducing retinal Müller gliosis [24]. Müller gliosis is generally regarded as the reactive proliferation of RMCs, therefore, RMCs were used to investigate the effects of EOFAZ on the morphological and functional changes of retina glia. Flow cytometry was used to investigate RMCs cycle. As shown in Fig. 3a, b, HG increased the percentage of RMCs in the S phase, implying that HG-induced RMCs proliferation. Pre-treatment with EOFAZ reversed the increase of S phase in a dose-dependent manner but did not affect the percentage of RMCs in the G1 and G2 phases. MTT assay shown that EOFAZ inhibited the abnormal proliferation of RMCs induced by HG (Fig. 3c). Giemsa staining shown that pre-treatment with EOFAZ significantly attenuated HG-induced abnormal morphology of RMCs (Fig. 3d). As shown in Fig. 3e–g, the transcription and expression of GFAP and VEGF are obviously enhanced in HG-treated RMCs, and EOFAZ negatively regulated GFAP and VEGF at the mRNA and protein level, As such, the HG-induced aberrant upregulation of GFAP and VEGF was inhibited. EOFAZ ameliorated HG-induced RMCs gliosis, and VEGF and GFAP were the targets of EOFAZ intervention.

### Effect of EOFAZ on PPAR-γ/CREB signaling in RMCs gliosis induced by HG

The selective activation of PPAR-γ exerts neuroprotection on retinal ganglion cells by suppressing reactive gliosis [13]. CREB, a nociceptive transcription factor, is phosphorylated by p-CaMK II at the onset of reactive gliosis [9]. Since the transcription of GFAP regulated by p-CREB, the relationship between EOFAZ and PPAR-γ/CREB signaling should be explored.

**Fig. 4:**
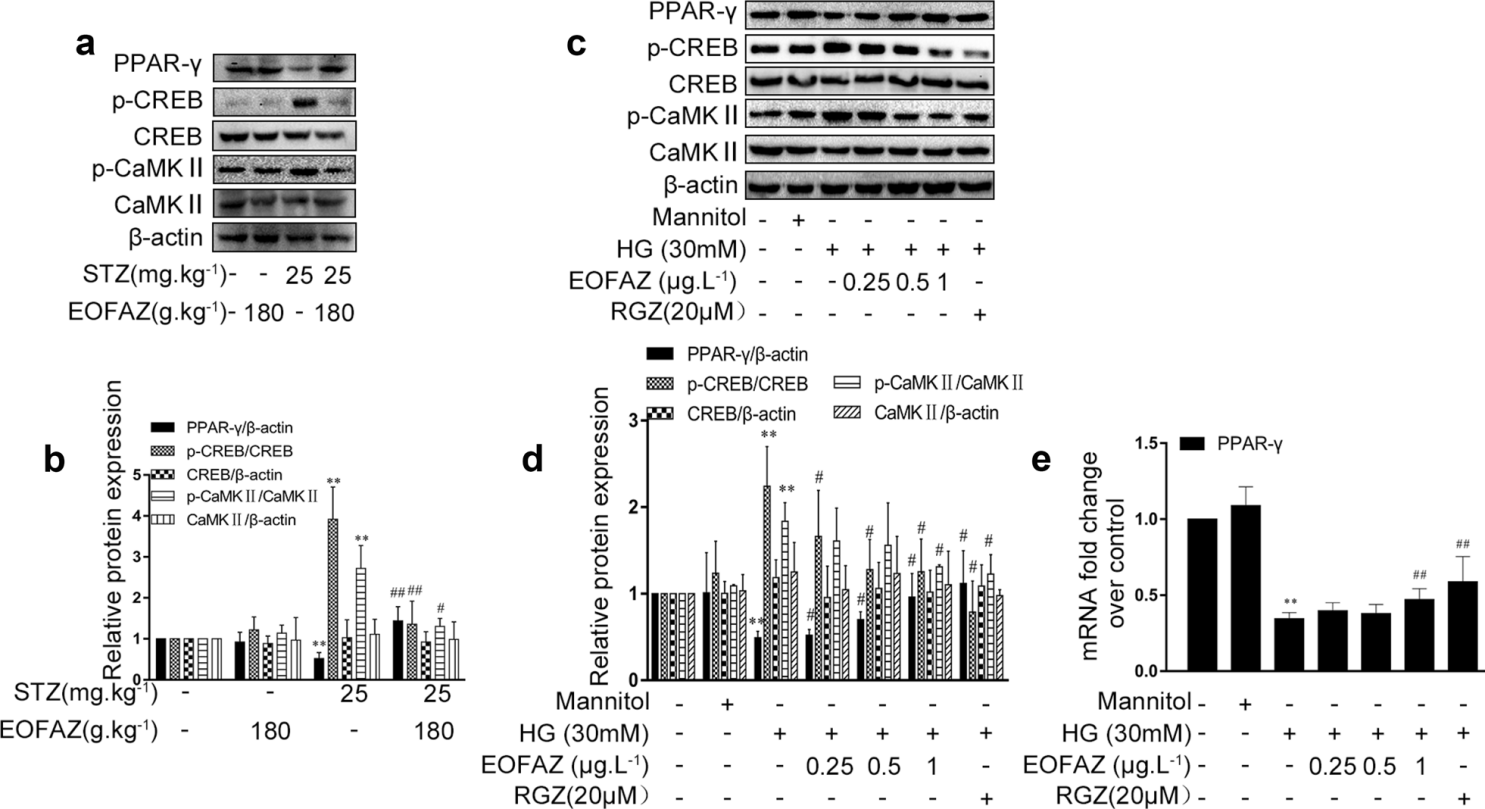
EOFAZ inhibited PPAR-γ downregulation and the high level of p-CREB and p- CaMKII in vivo and in vitro. Western blot analysis was used to analyze PPAR-γ, p-CREB (ser133), p-CaMK II (ser-286), total CREB and total CaMK II expression in rats (**a**, **b**). Western blots analysis was used to detect the expression of PPAR-γ, p-CREB (ser133), and p-CaMK II (ser-286), total CREB and total CaMKII levels in RMCs (**c**, **d**), mRNA level of PPAR-γ in RMCs detected by qRT-PCR (**e**). Data are expressed as mean ± SEM. n = 3. **P < 0.01 vs. non-diabetic rats or control group of RMCs, ^#^*P *< 0.05, ^##^*P *< 0.01 vs. Diabetic rats or HG group

The effects of EOFAZ on the expression of PPAR-γ, CREB, and CaMK II were investigated to determine whether the PPAR-γ/CREB signal was involved in EOFAZ amelioration on retinal gliosis. Western blot analysis revealed that CREB and CaMK II phosphorylation was obviously increased and PPAR-γ expression was significantly decreased in diabetic rats (Fig. 4a, b) and in RMCs challenged by HG (Fig. 4c, d). The high level of p-CREB, p-CaMK II and PPAR-γ downregulation was attenuated when rats or RMCs were treated with EOFAZ. qRT-PCR results further confirmed that EOFAZ could ameliorate HG-induced PPAR-γ mRNA reduction (Fig. 4e). In summary, EOFAZ could ameliorate PPAR-γ dysregulation and p-CREB signal in diabetic rat and HG-treated RMCs.

**Fig. 5:**
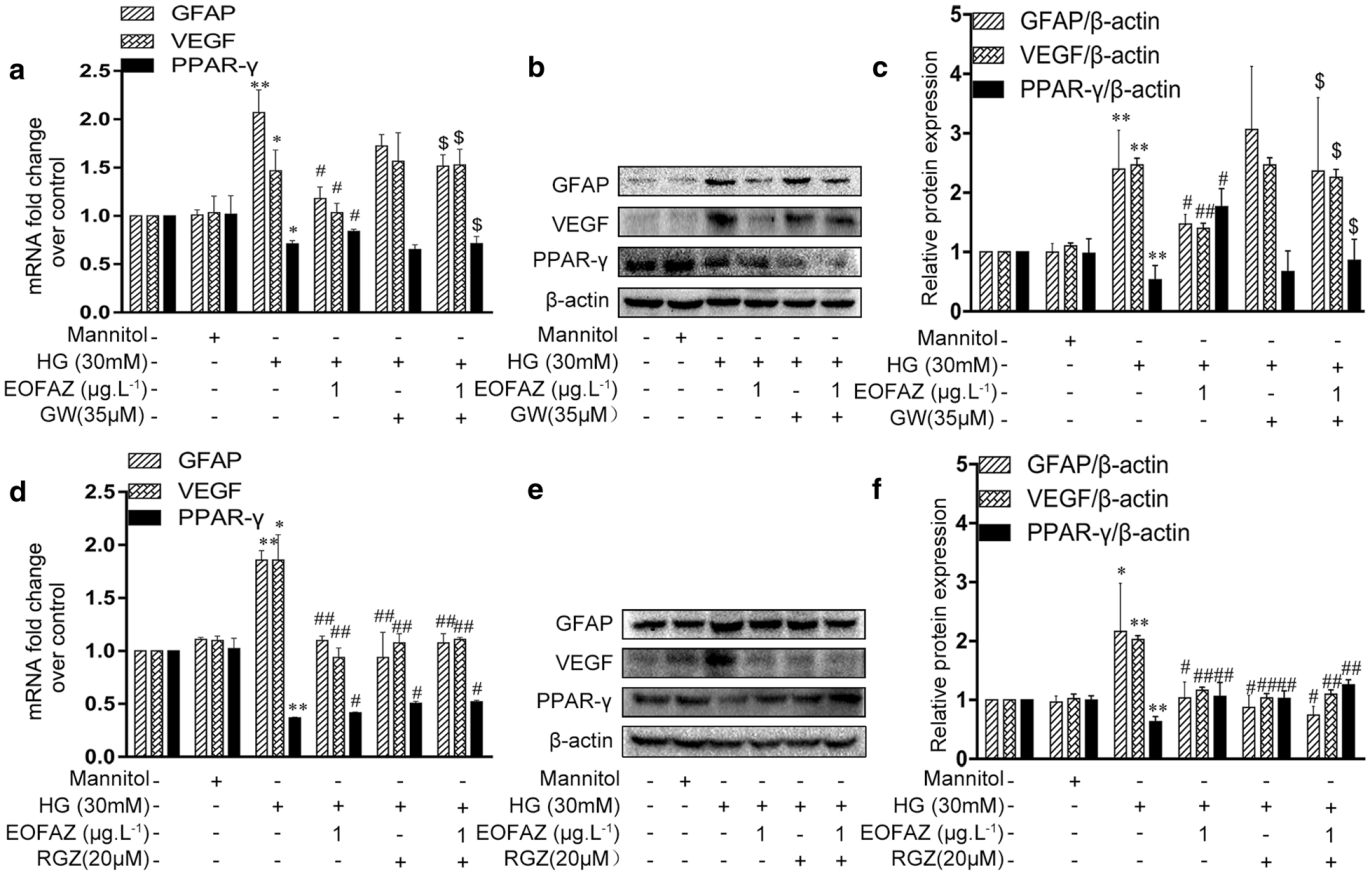
A PPAR-γ signal was involved in the inhibition effects of EOFAZ on RMCs gliosis induced by HG. GW9662 and RGZ were incubated alone or combined with EOFAZ in RMCs. The mRNA levels of GFAP, VEGF and PPAR-γ were detected via qRT-PCR (**a**, **d**). The protein expression of GFAP, VEGF and PPAR-γ was analyzed by Western blot analysis (**b**, **c**, **e**, **f**). Data are expressed as mean ± SEM. n = 3. *P < 0.05, **P < 0.01 vs. control group, ^#^*P *< 0.05, ^##^*P *< 0.01 vs. HG group, ^$^*P* < 0.05 vs.HG + EOFAZ group

The effects of PPAR-γ signal in EOFAZ-inhibiting retinal gliosis induced by HG were revealed by applying specific PPAR-γ agonist RGZ and PPAR-γ antagonist GW9662. EOFAZ inhibited the HG-induced upregulation of GFAP and VEGF but decreased the HG-induced downregulation of PPAR-γ, These effects could be considerably reversed by the action of GW9662. In addition, the difference in the GFAP, VEGF, and PPAR-γ transcription between the HG + EOFAZ and HG + EOFAZ + GW9662 groups was significant (Fig. 5a–c), suggesting EOFAZ ameliorated RMCs gliosis via activating PPAR-γ. RGZ enhanced the inhibitory effects of EOFAZ on the overexpression of GFAP and VEGF and the downregulation of PPAR-γ in HG-challenged RMCs (Fig. 5d–f).

**Fig. 6:**
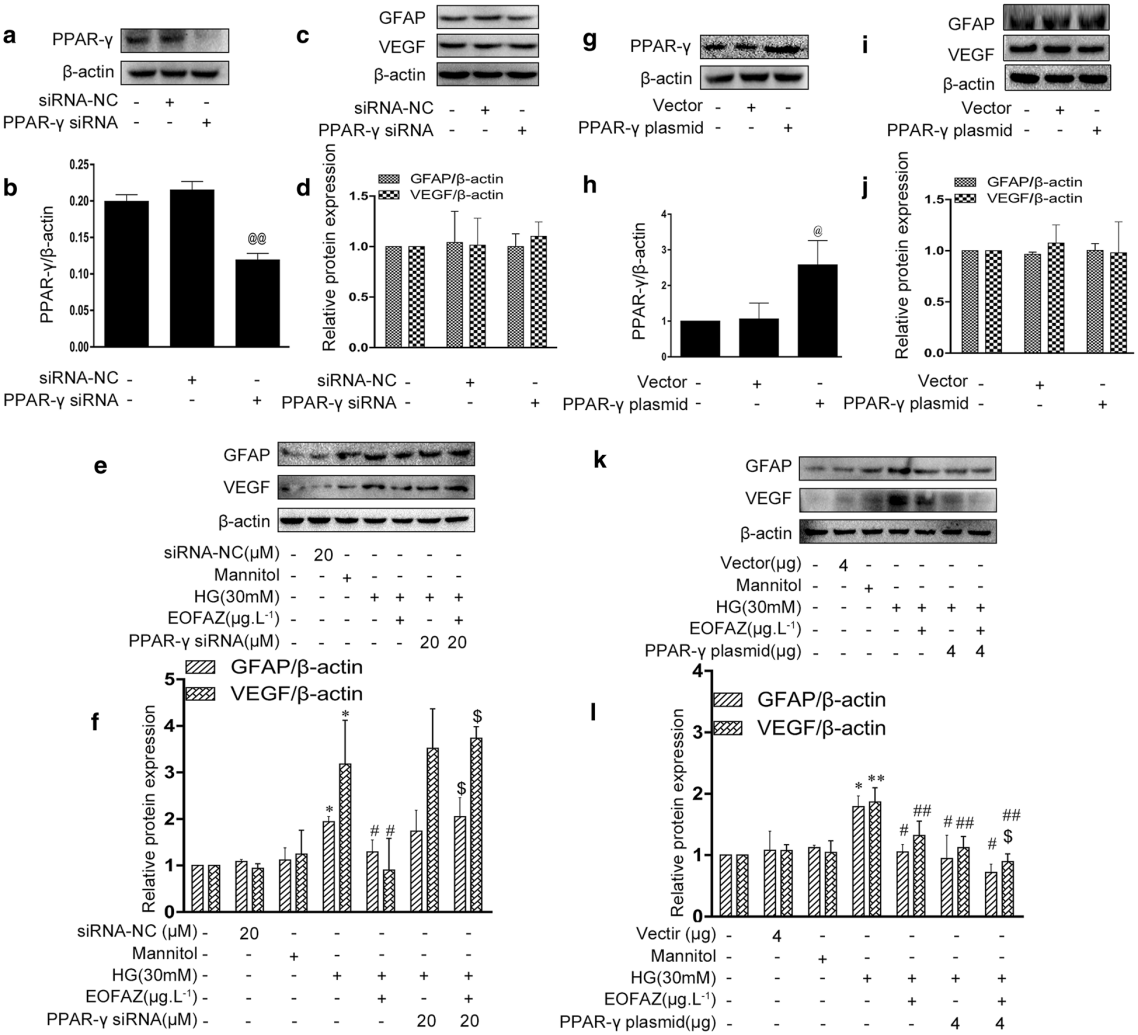
PPAR-γ signal is involved in the inhibition effects of EOFAZ on RMCs gliosis induced by HG. PPAR-γ siRNA (**a**–**f**) and PPAR-γ plasmid (**g**–**l**) were used alone or combined with EOFAZ in RMCs. PPAR-γ silencing efficiency of siRNAs (**a**, **b**). Study on PPAR-γ overexpression plasmid efficacy (**g**, **h**) and Western blot analysis was used to determine the protein expression of GFAP and VEGF (**c**, **d**, **i**, **j**. Western blot analysis was used to determine the protein expression of GFAP and VEGF (**e**, **f**, **k**, **l**). Data are expressed as mean ± SEM. n = 3. ^@^*P *< 0.05, ^@@^*P *< 0.01 vs. siRNA negative group or overexpression vector. *P < 0.05, **P < 0.01 vs. control group, ^#^*P *< 0.05, ^##^*P *< 0.01 vs. HG group, ^$^*P* < 0.05 vs.HG + EOFAZ group

PPAR-γ siRNA and overexpression plasmid were tested and applied to verify the effects of EOFAZ on PPAR-γ (Fig. 6). As shown in Fig. 6e, f, silencing PPAR-γ exerted a negligible effect on GFAP and VEGF overexpression. However, the protective effect of EOFAZ were abrogated in the presence of PPAR-γ siRNA. PPAR-γ overexpression and EOFAZ treatment reduced GFAP and VEGF overexpression. Moreover, the combined PPAR-γ plasmid transfection and EOFAZ treatment had a synergetic effect on inhibiting GFAP and VEGF overexpression (Fig. 6e–l). These results suggest that anti-gliotic property of EOFAZ partially depending on PPAR-γ activation.

**Fig. 7:**
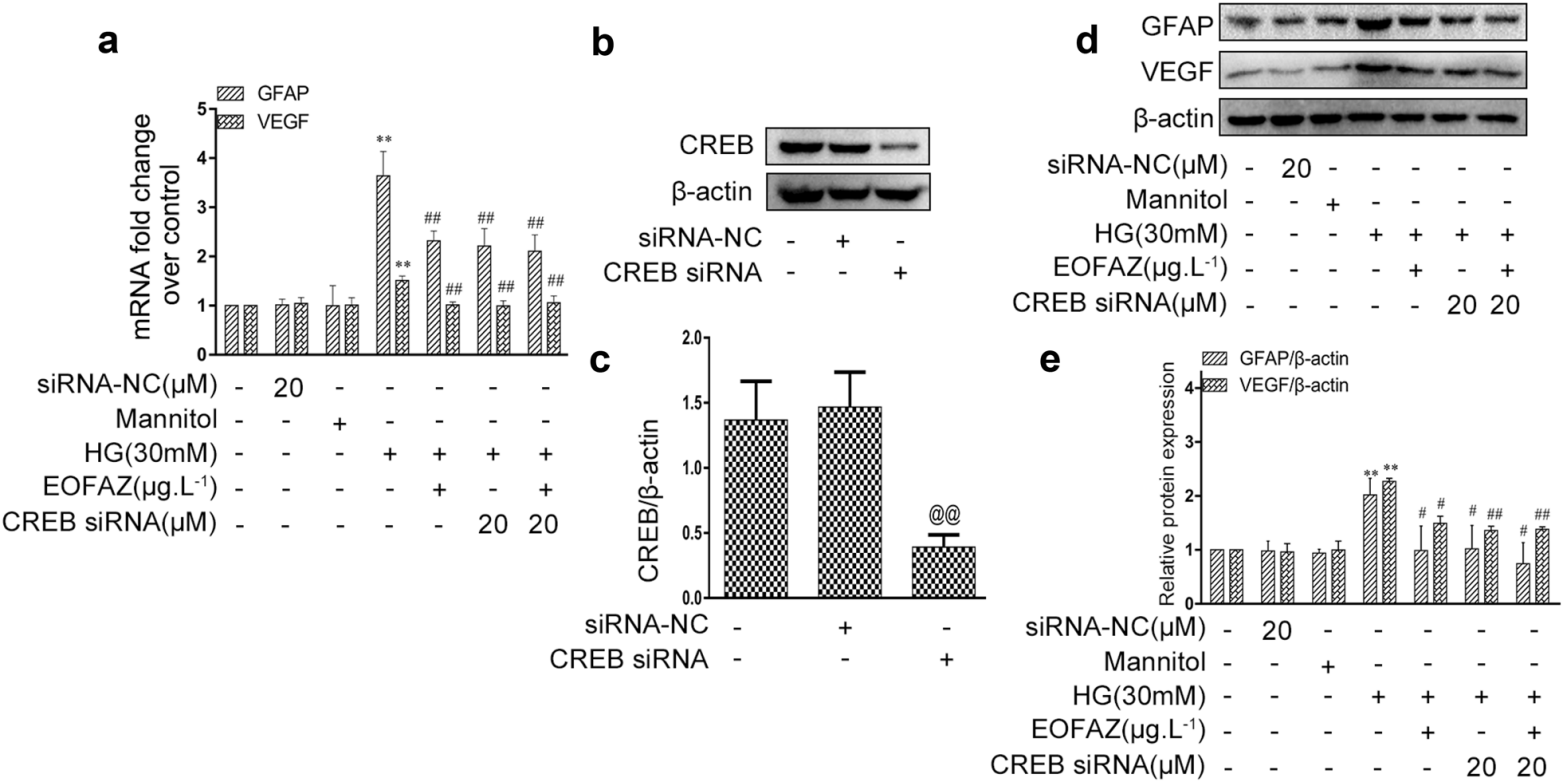
EOFAZ attenuated HG-induced RMCs gliosis by downregulating p-CREB. mRNA level of GFAP and VEGF in RMCs were detected by qRT-PCR (**a**). CREB siRNA silencing efficiency (**b**, **c**). Western blot analysis was used to determine the expression of GFAP and VEGF (**d**, **e**). Data are expressed as mean ± SEM. n = 3. ^@@^*P *< 0.01 vs. siRNA negative group. **P < 0.01 vs. control group, ^#^*P *< 0.05, ^##^*P *< 0.01 vs. HG group

CREB siRNA was used to explore the role of CREB in EOFAZ-inhibited RMCs gliosis. The results revealed that silencing CREB could significantly decrease GFAP and VEGF overexpression in the mRNA (Fig. 7a) and protein (Fig. 7d, e) levels. However, the difference in the expression of GFAP and VEGF protein between the HG + si-CREB and HG + si-CREB + EOFAZ group is not significant (Fig. 7d, e). Thus, the protective effects of EOFAZ are also related to p-CREB downregulation.

Overall, these results suggested that EOFAZ exerted protective effects on RMCs gliosis induced by HG at least partially via activating PPAR-γ and inhibiting phosphorylation of CREB.

### EOFAZ alleviated retinal Müller gliosis via the PPAR-γ-regulated p-CREB signal

**Fig. 8:**
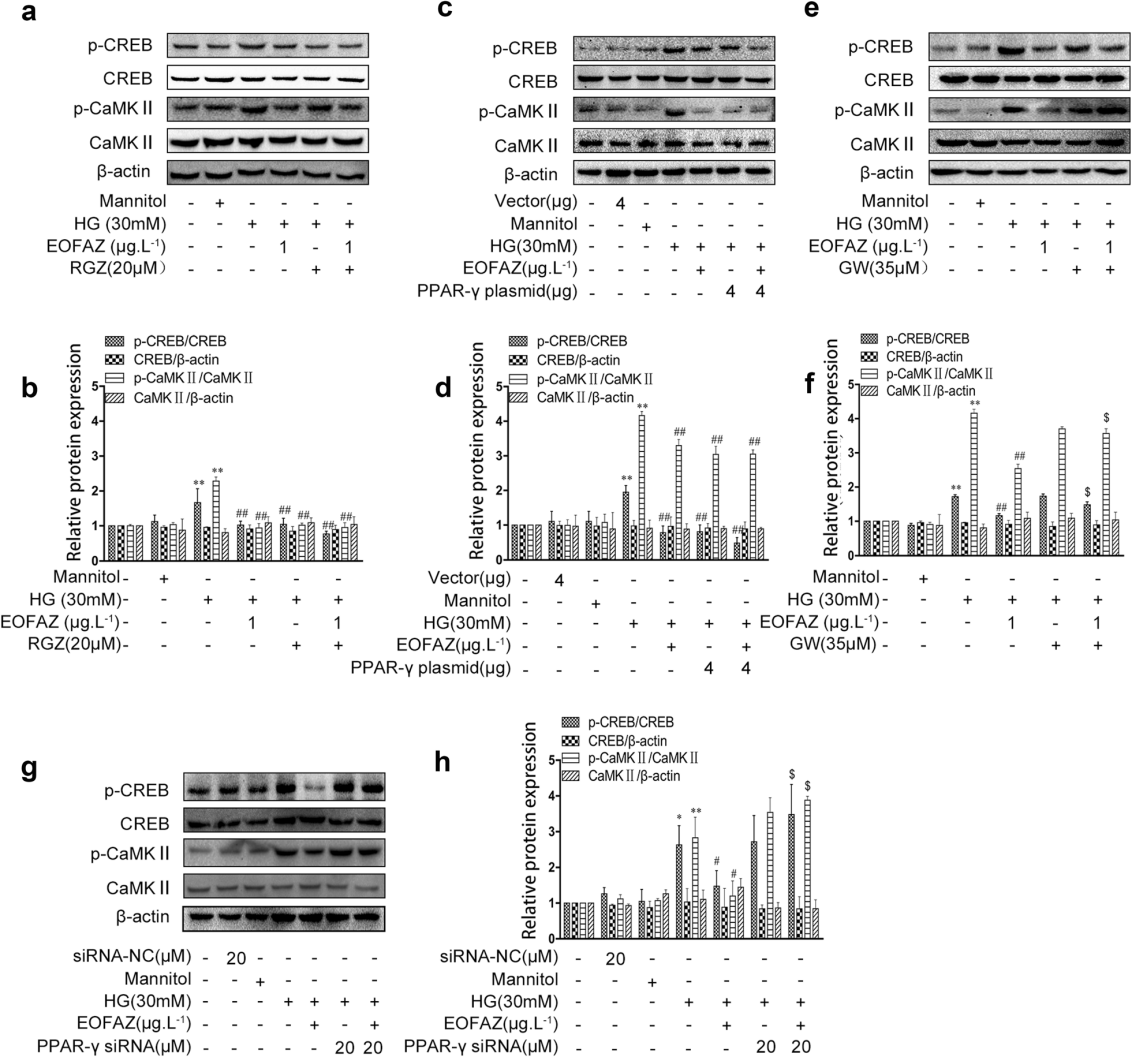
EOFAZ inhibited p-CREB and p-CaMKII in RMCs induced by HG via activating PPAR-γ. RGZ (**a**, **b**), overexpression of plasmid (**c**, **d**), and GW9662 (**e**, **f**) or PPAR-γ siRNA (**g**, **h**) were used alone or combined with EOFAZ in RMCs. The level of p-CREB and p-CaMK II and the expression of total CREB and CaMK II was detected via Western blot analysis. Data are expressed as mean ± SEM. n = 3. *P < 0.05, **P < 0.01 vs. control group, ^#^*P* < 0.05, ^##^*P *< 0.01 vs. HG group, ^$^*P* < 0.05 vs.HG + EOFAZ group

PPAR-γ inhibitors play a critical role in the transcriptional activation function of p-CREB [10], suggesting that PPAR-γ inhibition may mediate CREB signaling on retinal gliosis. Therefore, Western blot analysis was used to analyze the regulation PPAR-γ on p-CREB in retinal Müller gliosis. As shown in Fig. 8, PPAR-γ inhibition with GW9662 (Fig. 8e, f) or PPAR-γ silencing with siRNA (Fig. 8g, h) did not alleviate the HG-induced high level of p-CREB and p-CaMK II. The inhibitory effects of EOFAZ on p-CREB and p-CaMK II were abolished by additional treatment with GW9662 or PPAR-γ siRNA (Fig. 8e–h). The present data confirmed that PPAR-γ inhibition impaired EOFAZ negative modulation on retinal Müller gliosis and CREB and CaMK II phosphorylation.

Then, the PPAR-γ agonist RGZ and overexpressed PPAR-γ plasmid were employed in the following study, and the effects of activating PPAR-γ in reducing p-CREB and p-CaMK II were analyzed. As shown in Fig. 8a–d, EOFAZ, RGZ or PPAR-γ overexpression plasmid reduced the HG-induced phosphorylation of CREB and CaMK II, respectively. The combination of EOFAZ and RGZ treatment or PPAR-γ plasmid transfection suppressed p-CREB and p-CaMK II accumulation induced by HG. Thus, EOFAZ mitigates retinal Müller gliosis by activating PPAR-γ further reducing CaMK II and CREB phosphorylation.

**Fig. 9:**
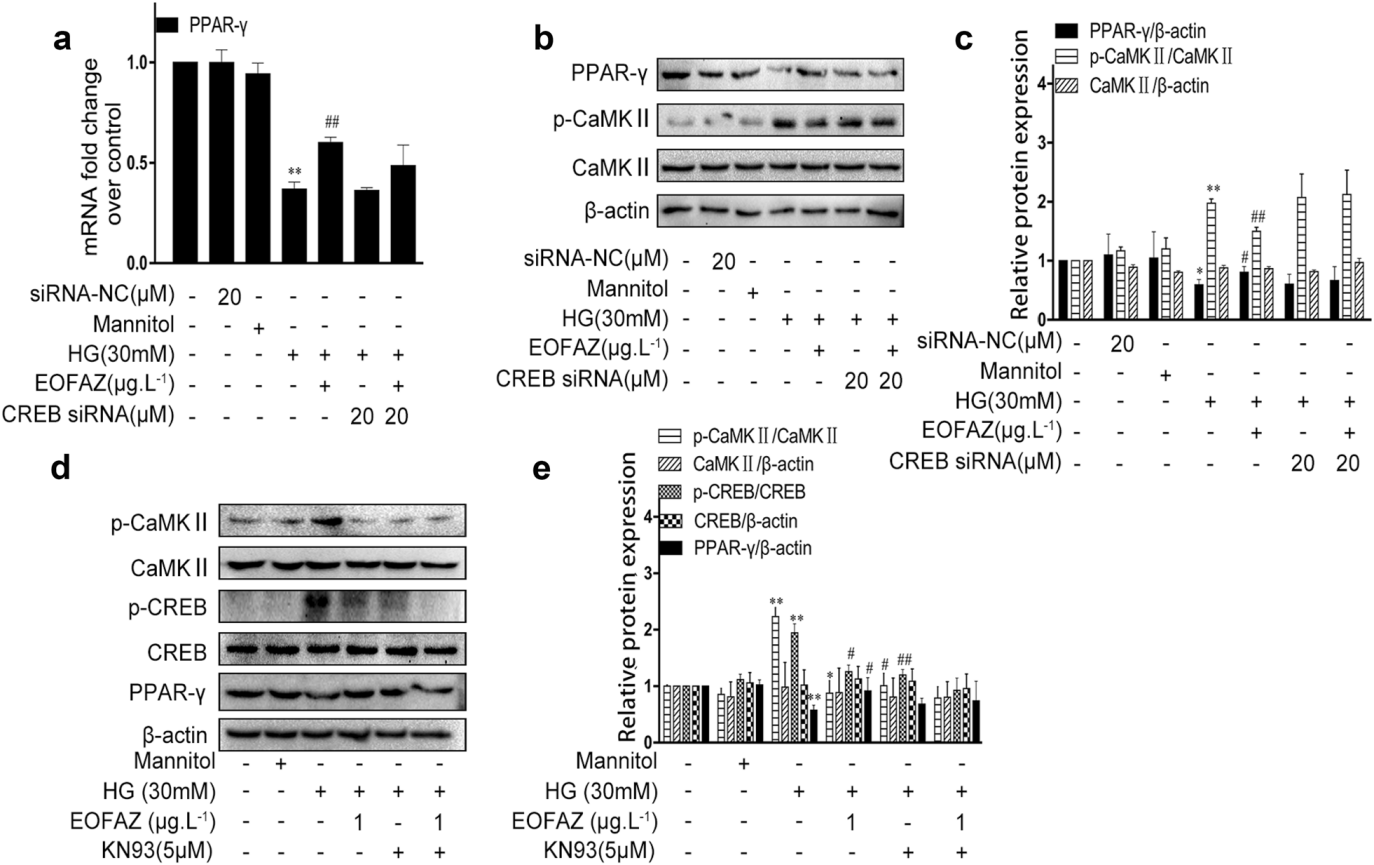
p-CaMKII mediated PPAR-γ-regulated p-CREB. qRT-PCR was used to detect the mRNA levels of PPAR-γ (**a**). The expression of PPAR-γ and CaMK II (**b**–**e**) in RMCs was determined via Western blot analysis. Data are expressed as mean ± SEM. n = 3. *P < 0.05, **P < 0.01 vs. control group, ^#^*P *< 0.05, ^##^*P *< 0.01 vs. HG group

Then, the effects of CREB-regulated PPAR-γ were analyzed. CREB siRNA had a negligible effect on PPAR-γ mRNA expression (Fig. 9a) and protein level (Fig. 9b, c). Silencing CREB did not change the expression of p-CaMK II (Fig. 9b, c). Those results suggested that PPAR-γ regulated p-CREB in RMCs gliosis induced by HG. KN93, a pharmacological inhibitor of CaMK II, was used in subsequent experiments to further validate whether the p-CaMK II-induced p-CREB was attributed to PPAR-γ inhibition in RMCs challenged by HG (Fig. 9d, e). KN93 treatment could significantly decrease the HG-induced phosphorylation of CaMK II and CREB, but slightly increase PPAR-γ expression when compared with the HG treatment group. Thus, p-CREB inhibition did not affect PPAR-γ activation.

The data show the protective effects of EOFAZ on retinal gliosis via the PPAR-γ-p-CaMK II-p-CREB signaling.

## Discussion

Diabetic retinopathy (DR) is a common complication of diabetes involving extensive damage to the retina [25]. Hyperglycemia is a major pathological factor responsible for the development of diabetic complications [26]. Various DR treatments have been developed, but all failed due to accompanying severe adverse effects [22], that affect their clinical applications. Therefore, novel therapeutic methods and targets in the prevention and treatment of DR must be developed. Considerable effort has been dedicated to developing new drugs for DR. One of the promising ones is an agonist of peroxisome proliferator activated receptors (PPARs) [27]. In our previous study, we found that EOFAZ activates PPAR-γ. Our current findings revealed the protective effects of EOFAZ against gliosis in the diseased retina and lay the foundation for EOFAZ as a complementary and alternative medicine for DR prevention and treatment in the future.

Alteration of the neuron-glia interaction is hightly associated with the development of neurodegenerative diseases [6, 28]. At present, the protective effects of EOFAZ on T_2_DM rats and HG-treated RMCs were investigated. The diabetic rat was reproduced via HFSD plus low dose of STZ injection. As shown in Fig. 1a–d, the changes in the body weight and blood glucose levels of diabetic rats were not obvious compared with those in non-diabetic ones when EOFAZ was administered for 8 weeks. This phenomenon is attributed to EOFAZ, which acts as a non-hypoglycemic agent capable of treating diabetic complications via unclear mechanisms.

The key events of DR are angiogenesis and easy hemorrhage into the pre-retinal space or the vitreous cavity, resulting in severe loss of vision [27]. The present results confirmed the occurrence of hemorrhage in the retina of diabetic rats, and EOFAZ ameliorated edema and hemorrhage in OPL and GCL. RMCs are distributed across the whole retina. The different effects of EOFAZ on ONL, OPL and INL remain unknown, and should be further examined in the future.

In response to pathological stimuli, Müller cells are reactivated, and undergo a variety of changes in cellular physiology, biochemistry and morphological features, called RMCs gliosis. RMCs gliosis is characterized by GFAP and VEGF upregulation [6, 29]. The abnormal upregulation of GFAP and VEGF was observed in diabetic rats (Fig. 2a, b), or HG-treated RMCs (Fig. 2d, e), respectively. The results are consistent with previous reports [5, 24]. The abnormal upregulation of GFAP and VEGF can be significantly ameliorated by treatment with EOFAZ, and treatment with EOFAZ also decreased the VEGF level in the serum of diabetic rats (Fig. 2c). Similar results were obtained in RMCs, as shown in Fig. 3a–d, EOFAZ protected against abnormal cell proliferation induced by HG. Giemsa staining revealed that EOFAZ can ameliorate the abnormal morphology of RMCs caused by HG (Fig. 3d). This study provides the first evidence that treatment with EOFAZ improved RMCs gliosis induced by HG in a dose dependent manner. Furthermore, EOFAZ could be a potential novel reagent for treating DR.

CREB activation at Ser133 is dependent on CaMK II phosphorylation, thereby inducing the expression of downstream genes, such as GFAP and VEGF. PPAR-γ has been developed as a major anti-diabetic drug target [25, 30]. The neuroprotective effects of PPAR-γ against reactive gliosis have been reported [13, 31]. Recent studies proposed that inhibiting PPAR-γ is involved in the activation of CREB, and precious regulatory mechanism of PPAR-γ activation and p-CREB suppression in drug treatment of retinal gliosis remains unknown.

As shown in Fig. 4, PPAR-γ expression in HG-treated RMCs and in the retina of the STZ + DM group rats was significantly lower than that in the control group, However, the level of p-CaMK II and p-CREB was considerably higher than that of the control group. EOFAZ treatment significantly increased the expression of PPAR-γ and decreased the level of p-CREB in RMCs and in the retina.

As shown in Figs. 5, 6, 7, PPAR-γ activation and p-CREB inhibition can reduce reactive gliosis by inhibiting the abrupt expression of VEGF and GFAP in RMCs. The main difficulty is identifying regulators of cross-talk between PPAR-γ- and CREB signals.Results showed that PPAR-γ downregulated CREB phosphorylation (Fig. 8). By contrast, silencing CREB hardly affected PPAR-γ activation (Fig. 9). Such results verify that PPAR-γ is the upstream of p-CREB. Also, PPAR-γ activation reduced CREB phosphorylation by suppressing p-CaMK II, the classical kinase responsible for CREB.

Thus, the present data support the hypothesis that the protective effects of EOFAZ on retinal gliosis are facilitated partly through CREB inhibition via PPAR-γ activation.

## Conclusion

**Fig. 10:**
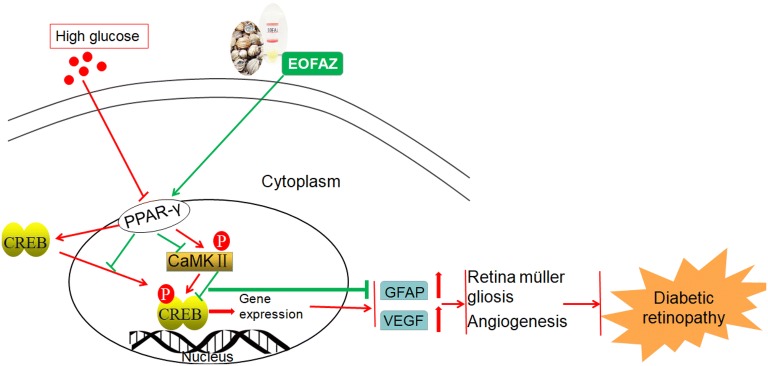
Scheme of mechanisms underlying the retinal protective effects of EOFAZ on DR. High glucose increases p-CaMK II-controlled p-CREB via the PPAR-γ inhibition. CREB activation stimulates GFAP and VEGF accumulation in the retina, thereby causing retinal glia gliosis and angiogenesis, and finally leading to DR. EOFAZ could attenuate the progression of DR by activating PPAR-γ and inhibiting CaMK II and CREB phosphorylation

EOFAZ exhibits protective effects on retinal gliosis partly mediated by PPAR-γ/p-CaMK II/p-CREB signaling (Fig. 10), thereby providing a novel theoretical basis for the clinical prevention and treatment of DR.

## Data Availability

The datasets used and/or analyzed during the current study are available from the corresponding author on reasonable request.

## Abbreviations

DR: diabetic retinopathy
HG: high glucose
EOFAZ: essential oil of Fructus *Alpiniae zerumbet*
RMCs: rat retinal Müller cells
VEGF: vascular endothelial growth factor
GFAP: glial fibrillary acidic protein
CREB: cAMP response element binding protein
PPARs: peroxisome proliferator-activated receptors
CaMK II: calcium/calmodulin depedent protein kinases II
RGZ: rosiglitazone
HFSD: high fat and high sucrose diet
TGs: serum triglycerides
TC: serum cholesterol
STZ: streptozotocin
HE: hematoxylin and eosin
HOMA-IR: homeostasis and insulin resistance index
